# Increased Conformity to Social Hierarchy Under Public Eyes

**DOI:** 10.3389/fpsyg.2021.636801

**Published:** 2021-07-15

**Authors:** Daeeun Kim, JuYoung Kim, Hackjin Kim

**Affiliations:** Laboratory of Social and Decision Neuroscience, School of Psychology, Korea University, Seoul, South Korea

**Keywords:** social dominance orientation, fear of negative evaluation, power, authority, observation, anonymity

## Abstract

Why would people conform more to others with higher social positions? People may place higher confidence in the opinions of those who rank higher in the social hierarchy, or they may wish to make better impressions on people of higher social status. We investigated how individual preferences for novel stimuli are influenced by the preferences of others in the social hierarchy and whether anonymity affects such preference changes. After manipulation of their social rank, participants were asked to indicate how much they liked or disliked a series of images. Then, they were shown the rating given to each image by a partner (either inferior or superior in social rank) and were given a chance to adjust their ratings. The participants were more likely to change their preferences to match those of a superior partner in the public vs. private condition. The tendency to conform to the views of the superior partner was stronger among those with higher social dominance orientation (SDO) and those with greater fear of negative evaluation (FNE) by others. Altogether, the findings suggest that the motivation to make better impressions on people of higher social status can be the major driver of conformity to others with higher social positions.

## Introduction

Most people belong to one or more groups that have a social hierarchy, and they often pay attention to social hierarchies within a group so as to succeed in social interaction, personal performance, and adaptation to the group (Chiao et al., 2004). People adjust their preferences, attitudes, and behaviors to conform to the norms of a group (Cialdini and Goldstein, 2004) as a means of benefiting their social and physical welfare within the group, because social hierarchy profoundly affects the welfare of both animals and humans (Sapolsky, 2004). Several recent studies of the behavioral and neural mechanisms of social conformity have shown that the preferences of individuals may be susceptible to contextual social information (Klucharev et al., 2009; Campbell-Meiklejohn et al., 2010; Izuma and Adolphs, 2013; Nook and Zaki, 2015). For example, preferences of participants for T-shirt designs changed toward those of a group they liked and away from those of a disliked group (Izuma and Adolphs, 2013). In addition, people consider it rewarding to find that their opinions match those of other people (Campbell-Meiklejohn et al., 2010), and they often change their decisions to align with the views of others so as to reduce incongruence (Klucharev et al., 2009).

Social conformity also depends on factors of power and hierarchy, in that individuals are more inclined to conform to the opinions and beliefs of people with higher status or ranking (Galinsky et al., 2008; Hays and Goldstein, 2015; Qi et al., 2018). For instance, Qi et al. (2018) found that people were more likely to conform to a high-reputation than a low-reputation collaborator when completing an uncertain perceptual task. These findings suggest that social conformity may be influenced by one’s knowledge about the relative differences in the hierarchy between oneself and others.

Why would people conform more to others with a higher social position? A possible explanation is that people are motivated toward greater accuracy by placing higher confidence in the opinions or decisions of those who rank higher in the social hierarchy (the accuracy hypothesis). Alternatively, they may wish to make better impressions on people of higher social status (the impression hypothesis). One way to investigate the underlying motivations for such social conformity is to manipulate the anonymity of choices of people. The impression hypothesis would predict that people will conform to those of higher social status only when their choices are visible to others, whereas the accuracy hypothesis would be supported if people conform to those of higher social rank regardless of the visibility of their choices. Previous studies have demonstrated that people are sensitive to the visibility of their decisions in conformity to group norms (Deutsch and Gerard, 1955), moral dilemmas (Lee et al., 2018), prosocial consumer decisions (Jung et al., 2018), charitable giving (Bereczkei et al., 2010), economic games (Haley and Fessler, 2005), and leadership matters (Case et al., 2018). However, to the knowledge of the authors, whether conformity to opinions of individuals with higher, but not lower, social hierarchy is affected by the anonymity of one’s choice is yet to be empirically demonstrated. Based on these findings, we hypothesized that people will be more likely to conform to others with higher social positions when they believe their choices are visible to them than when they believe that complete anonymity is guaranteed.

To test this hypothesis, we modified the social conformity task, in which participants have the opportunity to change their preferences so as to align with those of either of the two imaginary partners (Klucharev et al., 2009; Campbell-Meiklejohn et al., 2010; Izuma and Adolphs, 2013; Nook and Zaki, 2015). Importantly, the hierarchy of the confederates was ostensibly determined by their performances on a previous perceptual task (Hays and Goldstein, 2015). The participants made decisions in either a private or public condition at every trial. They were told that their choices in private conditions would not be revealed to the partners, but choices in public conditions would. The two colleagues were described as superior and inferior partners, and all the participants were assigned to an intermediate social rank between the two. In the main image preference rating task, the participants were asked to rate how much they liked or disliked each image and were then presented with either a congruent or an incongruent rating from the superior or the inferior partner for the same image (see “Materials and Methods” section for further details).

Given the prior evidence that the degree of social conformity varies substantially across individuals (Klucharev et al., 2009; Campbell-Meiklejohn et al., 2010; Izuma and Adolphs, 2013; Nook and Zaki, 2015), we also aimed to identify personal traits related to the individual differences in hierarchy-related conformity. To do so, we obtained responses to two scales, on social dominance orientation (SDO; Pratto et al., 1994) and fear of negative evaluation (FNE; Leary, 1983), in the debriefing questionnaires. Scores on the SDO scale are positively correlated with the motivation to seek higher social status. For example, people with higher SDO scores showed stronger motivation to maintain the legitimacy of the social hierarchy (Ligneul et al., 2017) as well as their power over others (Sidanius and Pratto, 2001), and they tended to rely more on social information (Cook et al., 2014). Based on these findings, we predicted that an increase in SDO would be associated with a higher likelihood of hierarchy-driven social conformity. Additionally, the FNE scale measures personality traits related to the apprehension about evaluations of others and the desire to pursue social approval (Leary, 1983). Previous studies have shown that FNE affects a variety of social behaviors, playing a particularly vital role in interpersonal interactions involving social evaluations (Wright et al., 2010; Van der Molen et al., 2014; Takagishi et al., 2016). Thus, people with a high level of FNE should be more likely to exhibit social conformity (Wright et al., 2010), especially in a public situation where their choices are visible to others.

Based on the previous literature and the *a priori* hypothesis described above, we sought to examine (1) whether the social hierarchy of others influences conformity of opinions, (2) whether such conformity due to social hierarchy is modulated by anonymity, and (3) whether individual differences in the degree of conformity due to social hierarchy can be predicted by results on the SDO and FNE scales.

## Materials and Methods

### Participants

The subjects were 60 Korea University students who had not participated in an independent image rating experiment previously (25 females, mean age = 23.7 ± 3.5 years). To determine the appropriate sample size for this study, we conducted an *a priori* power analysis with G^∗^Power 3.1.9.6 (Faul et al., 2007) based on the mean effect size of the interaction effects ($\eta_{p}^{2}=0.053$) that were drawn from four experiments in the previous study on power and conformity (see Study 2–5; Hays and Goldstein, 2015). The power analysis yielded that the required sample size at α = 0.05 with 95% power was *N* = 48, indicating that the sample size of this study is sufficient to detect a medium effect (Faul et al., 2007). Students majoring in neuroscience, psychology, or economics were excluded from participation, since they might have had prior familiarity with the behavioral task. The entire experimental procedure was approved by the Institutional Review Board of Korea University, and all experiments were performed in accordance with the relevant guidelines and regulations. Written informed consent was obtained from every participant before the behavioral experiment. All the participants were compensated with KRW 18,000 (=USD 15.5).

### Stimuli

One hundred twenty fractal images were prepared and used in the preference rating task. They were selected from a collection of 200 fractal images, which were evaluated by a separate group of 30 participants who rated how much they liked each image on a 4-point Likert scale from 1 (strongly dislike) to 4 (strongly like). Seventy fractal images with high percentages of either “strongly like” or “strongly dislike” ratings were excluded from the set of images used for the behavioral task, so as to better assess changes in preference.

### Experimental Procedure

Upon arrival at the laboratory, the participants were given an overall description of the experiment. Before participating in the first hierarchy manipulation task, they were informed that they would play an online game with two other participants located in separate rooms. The participants were given no information about the identities of the other participants. They were advised that they would participate in three activities: (1) social hierarchy manipulation, (2) preference rating task, and (3) modified dictator game. All activities were programmed and run using MATLAB (Mathworks Inc., Natick, MA, United States) and Cogent Graphics^1^ software packages.

#### Social Hierarchy Manipulation

A time estimation task and a visual discrimination task were used to manipulate the social hierarchy of the participants before the main task. Manipulating social hierarchy based on performance in a simple perceptual task has previously been shown to successfully engage participants in the manipulated social hierarchical context (Zink et al., 2008). The participants were informed that performance on these two perceptual tasks would determine their hierarchy in all the subsequent experiments. In reality, however, all the participants were assigned to the intermediate rank between their two imaginary partners. Both tasks were designed to be difficult to assess one’s performance, to ensure that the determined hierarchy would be believable.

**FIGURE 1:**
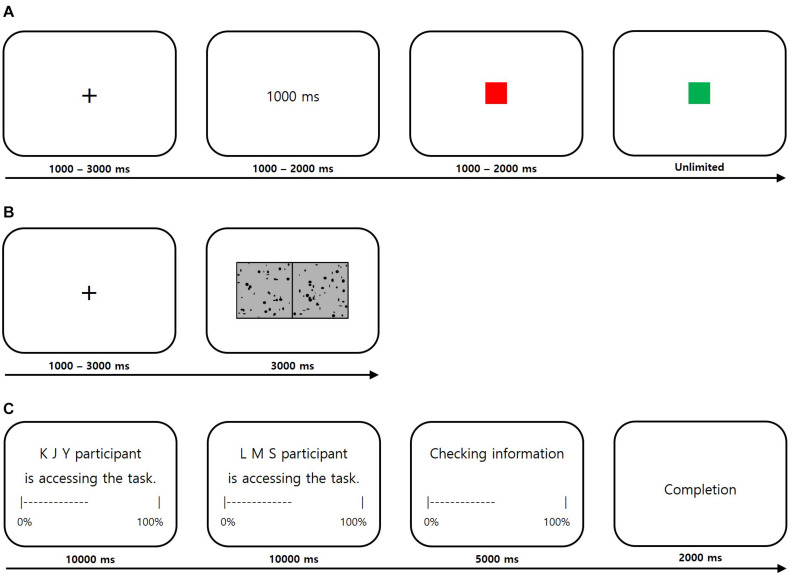
Schematic diagram of the behavioral tasks used in the social hierarchy manipulation. **(A)** The time estimation task. **(B)** The visual discrimination task. **(C)** Participants viewed that two other participants in separate laboratories were accessing an online game to maintain the believability of the interaction between each other.

First, in the time estimation task (Zink et al., 2008; Boksem et al., 2012; Figure 1A), the participants were instructed to respond precisely when a stipulated amount of time had passed after a signal. Each trial started with a fixation cross for 1–3 s, followed by the display of a goal time randomly selected from the time range between 100 and 2,000 ms. Then, a red square appeared as a ready signal for 1–2 s; when it turned green, the period of the time to be estimated began.

The second perceptual task was the visual discrimination task (Zink et al., 2008; Santamaría-García et al., 2014; Figure 1B). Each trial started with a fixation cross for 1–3 s, followed by the display for 3 s of randomly distributed black dots on a gray background, with a random number of 40–200 dots on one side and five more dots on the other side. The side with more dots was randomized across trials. The participants were instructed to indicate the side of the screen with more dots as soon as the dot screen appeared. If there was no response within 1 s, a message requesting an immediate response appeared at the bottom of the screen, and if another second passed with no response, the next trial began. The participants performed 30 trials of each task.

#### Preference Rating Task

**FIGURE 2:**
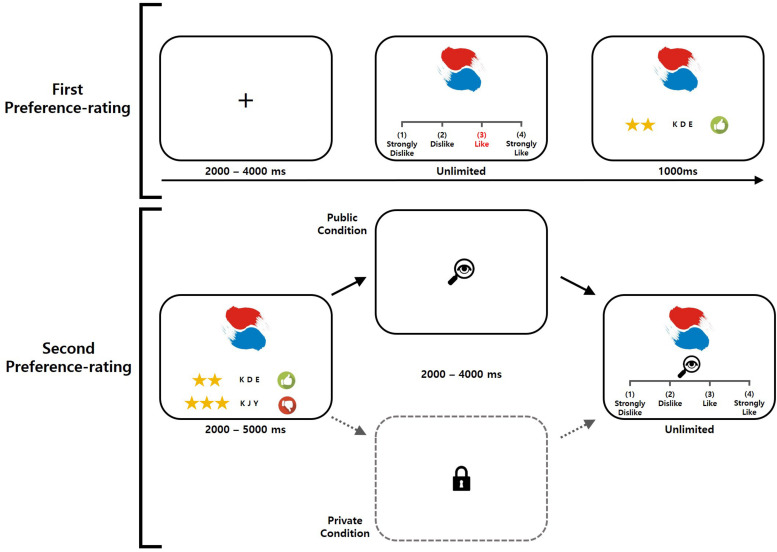
Schematic diagram of the preference rating task. Prior to the task, participants were informed that their final decisions in the public condition trials would be viewed by both their superior and their inferior immediately after the end of the preference rating task and before the modified dictator game. In contrast, participants were informed that in the private condition trials, their preference ratings would remain completely anonymous. Each trial of the task consisted of five distinct phases.

Following the social hierarchy manipulation, the participants were presented with a screen indicating that two other participants in separate laboratory rooms were accessing an online game (Figure 1C) to make them believe that they were interacting with real human partners. Each participant then performed the preference rating task (Figure 2), with 10 practice trials followed by the main task of 120 trials. Prior to the task, the participants were informed that their final decisions in the public condition trials would be viewed by both superior and inferior ranking partners immediately after the end of the preference-rating task. In contrast, their preference ratings would remain completely anonymous in the private condition trials.

Each trial consisted of five distinct phases. First, following the presentation of a jittered black fixation cross on a white background (for 2–4 s, with uniform distribution), a fractal image was presented above a 4-point Likert scale, and the participant was given an unlimited amount of time to evaluate and rate the image. Second, the rating of the participant was displayed visually with either a thumbs-down image (for a rating of 1 or 2) or a thumbs-up image (for 3 or 4) for 1 s. Third, the preference rating of either the superior or the inferior of the same fractal image was displayed for 2–5 s; and the ratings were determined such that congruent and incongruent ratings relative to the decision of the participant would be evenly distributed. Fourth, a symbol was shown to remind the participants that they were in either the public condition (an image of an eye in a magnifying glass) or the private condition (an image of a padlock) for 2–4 s. Finally, the same fractal image was shown again with a 4-point Likert scale, prompting the participant to evaluate and rate the image again, with an unlimited time permitted for responding.

#### Modified Dictator Game

The modified dictator game was included in the initial instructions, so as to emphasize and enhance the external validity of the hierarchy manipulation, but it did not actually take place. The participants were told that, after the main experiment, they would play a modified two-stage dictator game. In the dictator game, the superior partner would be given an endowment of $10 (i.e., approximately KRW 11,000) and told that he or she could allocate any fraction of it (ranging from $0 to $10) to the intermediate partner (i.e., all the participants in this study). Then, the intermediate partner could allocate any fraction of the remaining endowment to the inferior partner. For example, if the superior partner allocates $6 of the endowment for oneself, the intermediate partner gets $4, that is, the remainder of the endowment (i.e., $10 − $6 = $4). Next, if the intermediate partner allocates $3 of the remaining endowment for oneself, the inferior partner gets $1, that is, the rest of the endowment (i.e., $4 − $3 = $1). Therefore, after this game, the superior, intermediate, and inferior partner would be paid $6, $3, and $1, respectively. Inclusion of this game in the cover story was intended to let the participants informed of the structure of hierarchy and the asymmetry in power between them and the partners.

After completing all the tasks, the participants filled out the questionnaires measuring individual differences in personality, answered a few verbal interview questions, and then were debriefed about the deceptions regarding the absence of real human participants and the modified dictator game. Importantly, all the participants were asked to guess the purpose of the experiment, which was intended to check for possible demand characteristics, and none of them noticed the true purpose of the experiment, showing no suspicion of all the experimental manipulations.

### Post-experiment Questionnaires

The post-experiment questionnaires included the items from the SDO (Pratto et al., 1994) and FNE (Leary, 1983) scales. Higher SDO scores indicate a stronger drive to exercise domination over lower-ranked groups and greater preference for hierarchy within a social system (Pratto et al., 1994). The SDO questionnaire was composed of 16 items, such as “Some groups of people are simply inferior to other groups” and “We should do what we can to equalize conditions for different groups” (reverse-scored). The participants indicated their answers on a 7-point Likert scale (1 = strongly negative, 7 = strongly positive). The final SDO score was calculated by summing all the choices made by each participant. The overall SDO scores obtained in this study had adequate internal reliability (α = 0.81).

The FNE scale was used to measure one’s apprehension concerning possible negative evaluation by others (Leary, 1983), which we expected to be correlated with the effect of reputational concern in the public condition. The FNE scale consists of 12 items, such as “I worry about what other people will think of me even when I know it doesn’t make any difference” and “I am unconcerned even if I know people are forming an unfavorable impression of me” (reverse-scored). The participants indicated their ratings on a 5-point Likert scale (1 = strongly negative, 5 = strongly positive), and the overall FNE scores obtained in this study exhibited statistically significant internal reliability (α = 0.87).

### Behavioral Data Analyses

Across all trials, preference ratings were coded in a binary fashion, with ratings of 3 (“like”) and 4 (“strongly like”) coded as 1 and ratings of 1 (“strongly dislike”) and 2 (“dislike”) as 0 because the changes across the category better represents a conforming behavior, which is changing one’s belief or behavior to match that of others. Considering continuous changes would also be interesting, but in that case, changes within a category would be treated the same as the changes across categories. For instance, changing one’s preference rating from 1 (dislike very much) to 2 (dislike) and changing from 2 (dislike) to 3 (like) would both be changing 1 point on a Likert scale, but saying we dislike something less that we disliked more before may not be qualitatively the same as saying we like something that we disliked before. Then, the preference change in each trial was calculated by the absolute value of the difference between the first and second preference rating of the participant, which could range from 0 to 1.

For example, if a participant liked a given image during the first preference rating (coded as 1) and changed to disliking the same image in the second preference rating (coded as 0), the preference change for this trial would be | 1 – 0 | = 1. The mean preference change score for each condition was then entered into a 2 (social hierarchy: superior and inferior) × 2 (anonymity: private and public) × 2 (preference of partner: congruent and incongruent) repeated-measures analysis of variance (ANOVA). Paired-samples *t*-tests were carried out to examine the simple main effects that contributed to statistically significant interaction effects. We also ran a hierarchical multiple regression analysis to further examine any unique effect of SDO and FNE scores as well as the interaction effect on social conformity due to hierarchy and anonymity. Variance inflation factors were used to examine the presence of multicollinearity in the hierarchical multiple regression models, and no serious sign of multicollinearity was found in this study (VIF < 10) (Kutner et al., 2005). Moreover, we performed an additional analysis to verify whether the conformity tendencies are strongest for trials in which the discrepancy between the ratings of the participants and the ratings from the superior or inferior partner is largest. We first categorized the incongruent trials (i.e., 60 trials) based on how extreme the first preference of the participant for the image was, which would indicate different levels of incongruence between the initial preference of the participant and the preference of the partner. Thus, the trials where the participants rated 1 (i.e., preference of partner is 3 or 4) or 4 (i.e., preference of partner is 1 or 2) in the first rating were grouped as high-incongruence trials, and the trials where participants rated 2 (i.e., preference of partner is 3 or 4) or 3 (i.e., preference of partner is 1 or 2) were grouped as low-incongruence trials. The percentage of conforming behavior in each group of trials was calculated and entered into a paired-samples *t*-test. Post-experiment questionnaire data were normalized before further analyses. All behavioral analyses were conducted using the IBM SPSS Statistics (version 25) software.

## Results

### Increased Social Conformity Due to Social Hierarchy Under Public vs. Private Condition

**FIGURE 3:**
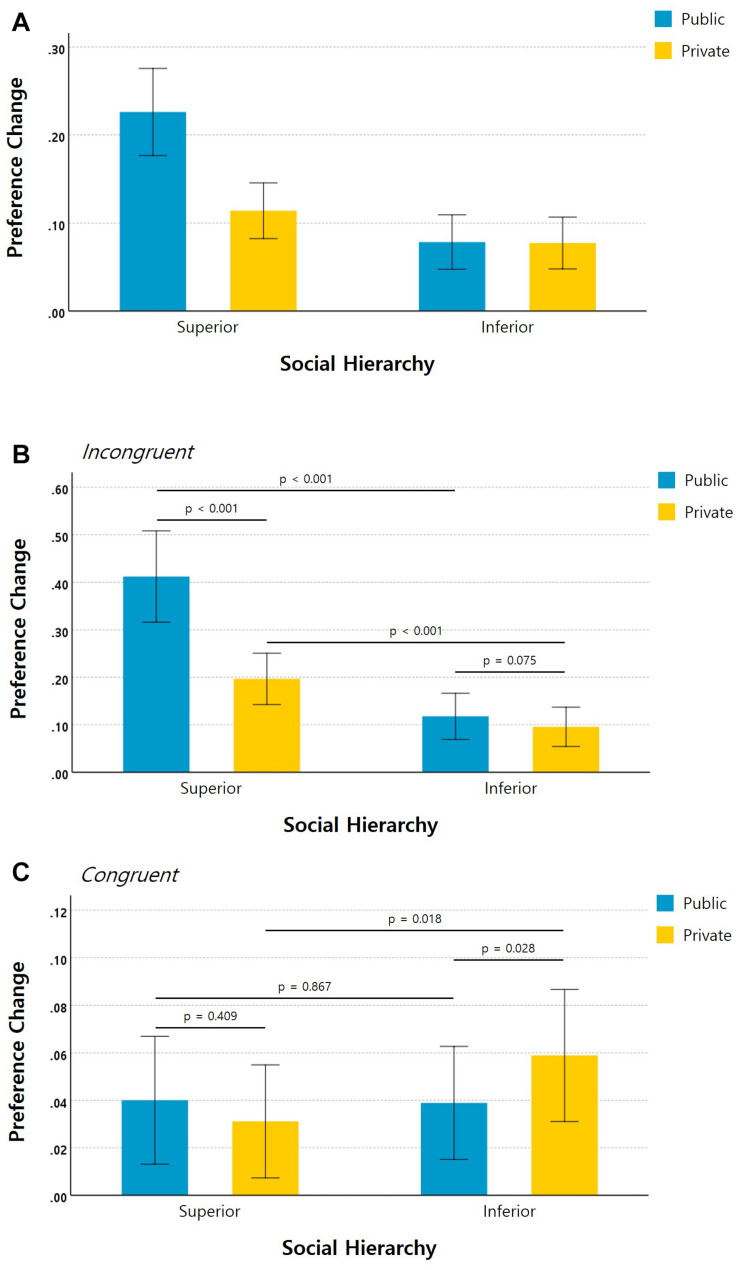
Preference changes due to social hierarchy and anonymity. **(A)** The degree to which participants changed their preferences from the first rating to the second rating was significantly influenced by the rating of the superior under the public condition. The same data are shown separately for **(B)** incongruent and **(C)** congruent preference trials (error bars indicate 95% confidence intervals).

The ANOVA results indicated that the changes in preferences of the participants regarding the fractal images were influenced by the social rank of the partner [*F*_(1, 59)_ = 27.48, *p* < 0.001, partial η^2^ = 0.32]; guarantee of anonymity [*F*_(1, 59)_ = 25.63, *p* < 0.001, partial η^2^ = 0.3]; and preference of the partner [*F*_(1, 59)_ = 54.31, *p* < 0.001, partial η^2^ = 0.48]. Moreover, this analysis yielded significant two-way interaction effects of social hierarchy × anonymity [*F*_(1, 59)_ = 30.55, *p* < 0.001, partial η^2^ = 0.34]; social hierarchy × partner’s preference [*F*_(1, 59)_ = 37.37, *p* < 0.001, partial η^2^ = 0.39]; and anonymity × preference of partner [*F*_(1, 59)_ = 33.94, *p* < 0.001, partial η^2^ = 0.37]. A significant three-way interaction effect on preference change was also significant [*F*_(1, 59)_ = 12.04, *p* = 0.001, partial η^2^ = 0.17]. *Post-hoc* analysis revealed that for the incongruent vs. congruent trials, the participants were more likely to change their preferences in line with those of the superior vs. inferior partner in the public vs. private condition (Figure 3A).

*Post-hoc* paired-sample *t*-tests were conducted to assess the behavioral responses between each pair of conditions. In the incongruent condition, the participants were more likely to change their preferences in line with those of the superior (*M* = 0.41, *SD* = 0.37) rather than the inferior partner (*M* = 0.12, *SD* = 0.19) under the public condition [*t*_(__59__)_ = 5.78, *p* < 0.001, Cohen’s *d* = 1]. They were also more inclined to align their preference with that of the superior (*M* = 0.2, *SD* = 0.21) than with that of the inferior (*M* = 0.1, *SD* = 0.16) in the private condition [*t*_(__59__)_ = 4.04, *p* < 0.001, Cohen’s *d* = 0.54]. These results also indicate that the participants were more likely to conform to the opinion of the superior in the public condition (*M* = 0.41, *SD* = 0.37) than in the private, anonymous condition (*M* = 0.2, *SD* = 0.21) [*t*_(__59__)_ = 5.42, *p* < 0.001, Cohen’s *d* = 0.71]. In contrast, when shown the decision of the inferior, the degree of conformity of the participants showed no significant difference between the public (*M* = 0.12, *SD* = 0.19) and private conditions (*M* = 0.1, *SD* = 0.16) [*t*_(__59__)_ = 1.82, *p* = 0.075, Cohen’s *d* = 0.13] (Figure 3B).

For the congruent condition, there was no significant difference in effect between the decisions of the superiors (*M* = 0.04, *SD* = 0.1) and the inferiors (*M* = 0.04, *SD* = 0.09) under the public condition [*t*_(__59__)_ = 0.17, *p* = 0.867, Cohen’s *d* = 0.01]. However, the participants were more likely to change their decisions in opposition to those of the inferior (*M* = 0.06, *SD* = 0.11) than the superior partner (*M* = 0.03, *SD* = 0.09) under the private condition [*t*_(__59__)_ = 2.42, *p* = 0.018, Cohen’s *d* = 0.28]. Furthermore, when the participants were presented with the rating from the superior partner, there was no significant difference between their behavior in the public (*M* = 0.04, *SD* = 0.1) and private conditions (*M* = 0.03, *SD* = 0.09) [*t*_(__59__)_ = 0.832, *p* = 0.409, Cohen’s *d* = 0.09]. On the other hand, when the rating of the inferior was presented, the subjects were more likely to adjust their rating in the opposite direction from the inferior under the private (*M* = 0.06, *SD* = 0.11) than under the public condition (*M* = 0.04, *SD* = 0.09) [*t*_(__59__)_ = 2.26, *p* = 0.028, Cohen’s *d* = 0.2] (Figure 3C).

Furthermore, a paired-samples *t*-test was conducted to investigate the degree of conformity effect. The analysis revealed no significant difference in conformity effect between low-incongruence trials (*M* = 0.23, *SD* = 0.18) and high-incongruence trials (*M* = 0.18, *e* = 0.25) [*t*_(__59__)_ = 1.84, *p* = 0.071, Cohen’s *d* = 0.23].

### Individual Differences in Social Dominance Orientation Moderating Social Conformity, and the Effect of Hierarchy and Anonymity

**FIGURE 4:**
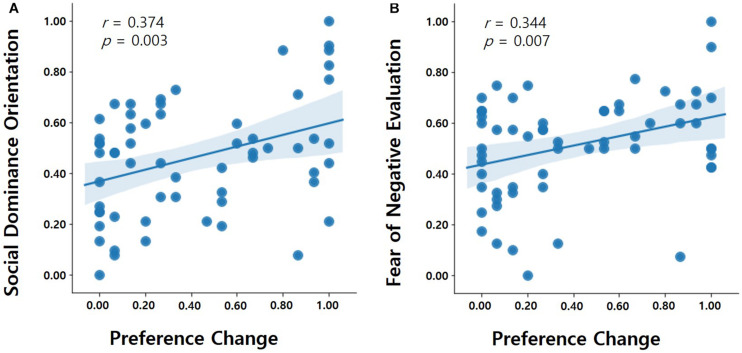
Individual differences predicting the degree of social conformity. The degree to which participants changed their preferences in incongruent trials due to social hierarchy under the public condition were predicted by individual differences in **(A)** the normalized SDO scores (*r* = 0.37; *p* = 0.003, two-tailed Pearson correlation) as well as by **(B)** the normalized FNE scores (*r* = 0.34; *p* = 0.007, two-tailed Pearson correlation).

We then examined whether any personality traits were associated with the individual differences in social conformity due to hierarchy and anonymity. To do so, we performed correlation analyses to evaluate the associations between each measurement (i.e., the SDO and FNE) and preference changes of the participants in the public condition. A significant positive correlation was observed between the normalized SDO scores and preference changes of the participants [*r*_(__60__)_ = 0.37, *p* = 0.003, two-tailed Pearson correlation] (Figure 4A), as well as between the normalized FNE scores and preference changes of the participants [*r*_(__60__)_ = 0.34, *p* = 0.007] (Figure 4B). In the private condition, however, the degree of conformity to superior partners was not associated with SDO [*r*_(__60__)_ = 0.02, *p* = 0.906] and FNE [*r*_(__60__)_ = 0.1, *p* = 0.448].

To further examine the unique effects of SDO and FNE scores as well as the interaction effect on social conformity due to hierarchy and anonymity, we also ran a hierarchical multiple regression analysis. In the first step of the hierarchical multiple regression, SDO and FNE were entered as predictor variables, and the degree of social conformity (represented by the preference change in the public condition) was entered as the criterion variable. The results showed that Model 1 was significant [*F*_(2, 57)_ = 7.54, *p* = 0.001], and accounted for 21% of the variance (*R*^2^ = 0.21). The degree of social conformity was uniquely associated with one’s SDO (β = 0.31, *p* = 0.013) and FNE (β = 0.27, *p* = 0.029).

In the second step of the hierarchical multiple regression, the interaction between the two scale scores (i.e., SDO × FNE) was added to the regression model of the first step. The result of Model 2 indicated that no significant interaction effects emerged [*R*^2^ change = 0.001, *F*_(1, 56)_ = 0.041, β = −0.079, *p* = 0.84] (Table 1). Therefore, both SDO and FNE, but not their interaction, were unique and significant predictors of social conformity to hierarchy in the public condition.

**TABLE 1:** Significant predictors of social conformity in hierarchical multiple regressions (*N* = 60).

Model	*R*^2^	*Adj.R^2^*	*R^2^-*change	*F*-change	*DF*	*p*
Model 1	0.209	0.182	0.209	7.452	2.57	0.001
Model 2	0.210	0.167	0.001	0.041	1.56	0.840

**Variable**	***B***	***SE B***	**β**	***t***	***p***

**Model 1**					
SDO	0.507	0.198	0.310	2.557	0.013
FNE	0.500	0.223	0.272	2.242	0.029
**Model 2**					
SDO	0.592	0.467	0.362	1.268	0.210
FNE	0.580	0.456	0.315	1.272	0.209
SDO°×°FNE	−0.157	0.777	−0.079	−0.202	0.840

We performed the same correlation analyses for the private condition. Unlike the results for the public condition, the tendency of the participants to differentiate themselves from the inferior in congruent trials under the private condition was associated with neither SDO [*r*_(60)_ = −0.02, *p* = 0.897] nor FNE [*r*_(__60__)_ = −0.12, *p* = 0.345]. Furthermore, there was no association between the tendencies to differentiate oneself from the inferior in congruent trials and to conform to the superior in incongruent trials under the public condition [*r*_(__60__)_ = 0.08, *p* = 0.55].

## Discussion

This study aimed to investigate changes in preference caused by the visibility of one’s own choices and opinions of others of different social status. Based on previous studies and theory, we hypothesized that social conformity would be affected by social hierarchy, especially when one’s decisions are publicly known. We adopted and modified the social conformity paradigm used in previous studies (Galinsky et al., 2008; Klucharev et al., 2009; Izuma and Adolphs, 2013) to test the hypothesis. The results showed that the participants were more likely to change their preferences in line with those of the superior partner and in the public rather than in the private condition. In other words, they are more inclined to publicly conform to those with the authority in the hierarchical situation (i.e., the modified dictator game) or with expertise (i.e., a higher performance in the perceptual tasks) than unauthorized partner or non-expert. In addition, this increased social conformity due to hierarchy and publicity was moderated by individual differences in SDO and FNE. Taken together, the findings provide evidence supporting a useful framework for understanding social conformity where people who are more oriented toward social dominance tend to follow the opinions of those with higher social status particularly when their decisions are visible to those who hold power.

Previous studies of social conformity have shown that social behaviors may be influenced by one’s knowledge about the relative differences in status or rank between oneself and others (Galinsky et al., 2008; Hays and Goldstein, 2015; Qi et al., 2018). For example, when people submit a proposal to their boss, they may want to align their beliefs, attitudes, and behaviors with those of the boss, to facilitate acceptance and gain social approval (Cialdini and Goldstein, 2004). However, it has not been clear whether such social conformity occurs, because people tend to believe that the opinions of the boss are more accurate and better informed or because they fear the disapproval of the boss. Extending these previous findings, this study further reveals that social conformity induced by differences in the social hierarchy may also be modulated by guaranteed anonymity. In other words, people are less likely to exhibit social conformity to their superiors when they believe that their decisions and opinions will remain anonymous. Therefore, avoiding fear of social disapproval by those with higher social power could be a powerful motivation for social conformity in this context.

We further explored how individual differences with regard to SDO and FNE by others affect or bolster social conformity. We found that social conformity to the superior partner, especially in public social situations, was higher among people with high SDO and also among those with high FNE. More specifically, participants with higher SDO were more likely to conform to opinions of superiors when they believed that their decisions would be revealed to the partner. High SDO has been empirically shown to be associated with preferences for hierarchy within a social group and with dominant behaviors relative to lower-status people (Sidanius and Pratto, 2001; Ligneul et al., 2017). Individuals with higher SDO scores should be more motivated to align their opinions with those of their superiors, because of their higher sensitivity to social hierarchy and their increased tendency to conform to authorities or those in power. Importantly, however, this study indicated that SDO was not associated with conformity to superior partners, when the participants believed that anonymity was guaranteed. This result is consistent with the empirical evidence that people are more likely to display socially desirable behaviors when they are visible to other people (Haley and Fessler, 2005; Bereczkei et al., 2010; Case et al., 2018; Jung et al., 2018; Lee et al., 2018). In view of these findings, conformity of the participants to opinions of superiors appears to be a strategic move to satisfy the motivation to seek social dominance, which may be triggered when their behaviors are visible to people with high social rank. Given that the mean SDO score in the data (*M* = 53.02, *SD* = 11.84) was higher than other samples in European and Western societies (Fischer et al., 2012), however, it is left answered whether the main findings, such as the relationship between SDO and conforming behavior, in social hierarchical context would be replicated in other (i.e., European and Western) cultures.

Based on the evidence that social conformity is often associated with the motivation to seek approval from others (Cialdini and Goldstein, 2004), we also measured the FNE of the participants (Leary, 1983) to investigate whether individual differences in the apprehension of negative evaluations by others and the desire to seek social approval would be associated with the degree of social conformity exhibited. As predicted, the participants with higher FNE scores were more inclined to show increased social conformity to superior partners in the public condition. Previous research has shown that people with high FNE are particularly likely to display conformity (Wright et al., 2010), fear the loss of social approval (Watson and Friend, 1969), and be more socially anxious (Leary, 1983). Furthermore, from an evolutionary perspective, a high inclination to avoid negative social evaluation by others can be expected to have evolved into a biologically more suitable trait to avoid potential interpersonal conflicts with individuals who rank higher in the social hierarchy (Gilbert, 2001). Based on these empirical and theoretical insights, we can infer that people who fear the negative evaluation of others will be particularly attentive to the opinions of superior partners and more likely to conform to the preference of their superior under the public condition, because such conformity would meet their need to seek social rewards (Klucharev et al., 2009; Izuma, 2017). It should be noted, however, that this finding is only correlational rather than causational.

Another interesting finding in this study is that the participants showed a tendency toward negative social influence; that is, they adjusted their decisions away from opinions with dominant people of inferiors in the private condition. This appears to be in line with a previous finding that preferences of college students for T-shirts they initially liked decreased after they learned that the sex offenders liked the same items (Izuma and Adolphs, 2013). However, the investigation into the impact of either SDO or FNE on the degree of negative social influence from inferiors in the private condition revealed no meaningful association. Given that this negative social influence is not associated even with social conformity to superiors in the public condition, it may be grounded in a distinct but weaker motivation that also contributes to the overall effect of social hierarchy and publicity on social conformity. According to the balance theory (Heider, 1946), the fact that one has the same opinion as an inferior may cause psychological discomfort, and changing one’s opinion may thus be a means to relieve such discomfort. Considering that such negative social influence was higher in the private than in the public condition, the motivation to follow the opinion of the other person regardless of the social hierarchy may have offset the motivation to avoid holding the same opinion as an inferior. Although this interpretation may be plausible, further research is needed to explore the motivation for this negative influence of social hierarchy on social conformity.

A few limitations regarding the experimental paradigm should be considered carefully in interpreting the results. First, although no participant reported any other specific motivation behind the conforming behavior in the in-depth interview during the debriefing session, we cannot entirely exclude the possibility that the experimental manipulation of social hierarchy could have elicited motivations other than making a better impression on the higher status partner. For example, people might be more inclined to publicly conform to those with more expertise (i.e., a higher performance in the perceptual tasks) than non-experts. This alternative possibility could be tested at least partly by comparing the results of this study with those from a separate study where the instruction about a dictator game is omitted from the cover story. Moreover, because of the absence of a non-social condition, this research design does not allow us to conclude whether the observed conformity was driven purely by social motivation. Therefore, future research may be necessary to rule out all these alternative interpretations for the motivation behind conformity observed in this study. Second, it should be noted that the within-subjects design used here could have made it easier for the participants to be influenced by the explicit disparity between the high- and low-hierarchy partners. We believe that the chance of demand characteristic is low because no participant noticed the real purpose of the study and the individual traits in SDO and FNE correlated with the selective conformity to higher vs. lower status partners under public condition, although we should be cautious in interpreting the results. Additionally, we did not perform an *a priori* power analysis to determine the required sample size for the correlation analysis, because there are no previous studies that used the same analysis. Therefore, we conducted *post-hoc* power analyses with G^∗^Power 3.1.9.6 (Faul et al., 2007) on the correlation analyses between SDO or FNE scores and conformity to higher status partner under public eyes. The achieved power with the 60 participants at α = 0.05 for the correlations of conformity with SDO and FNE was 84% (the effect size = 0.37) and 77% (the effect size = 0.34), respectively. The recommended sample sizes for SDO and FNE to achieve the power of 95% of this analysis were *N* = 86 and *N* = 103, respectively. Therefore, we suggest that the result with FNE be considered as exploratory, and a larger sample size may be necessary to verify the role of FNE in conformity to higher status under public eyes.

In sum, this study demonstrates that people are more likely to change their initially discrepant views and conform to those of superiors than inferiors, especially when they believe that their opinions will be revealed to their colleagues. Also, the degree of social conformity due to hierarchy and publicity is greater among those with higher SDO, as well as among those with higher FNE. The results suggest that successful navigation of concerns for social conformity (as reflected in preference changes to match the views of superiors) could be influenced not only by the ability of one to recognize the relative social hierarchy of others but also by reputational concerns. To extend the existing body of literature on the neural basis of social conformity (Klucharev et al., 2009; Campbell-Meiklejohn et al., 2010; Izuma and Adolphs, 2013; Nook and Zaki, 2015; for a meta-analysis, see Wu et al., 2016), a future neuroimaging study could investigate the neural mechanisms whereby social hierarchy and anonymity modulate social conformity. Such a study would further expand the understanding of how the brain incorporates various types of social information and prioritizes only a subset of this information to achieve efficient decision-making in a complex social environment.

## Data Availability Statement

The original contributions presented in the study are included in the article/supplementary material, further inquiries can be directed to the corresponding author/s.

## Ethics Statement

The studies involving human participants were reviewed and approved by the Institutional Review Board of Korea University. The patients/participants provided their written informed consent to participate in this study.

## Author Contributions

HK and DK conceived of the study. HK, DK, and JK designed experimental procedures, performed the data analyses, and wrote the manuscript. DK conducted the experiment and collected the data. All authors read and approved the final manuscript.

## Conflict of Interest

The authors declare that the research was conducted in the absence of any commercial or financial relationships that could be construed as a potential conflict of interest.

## Footnotes

1 http://www.vislab.ucl.ac.uk/cogent.php
